# Use of Hoteling’s T^2^ multivariate control chart for effective monitoring of a laboratory test with a 3-level quality control scheme

**DOI:** 10.11613/BM.2025.020701

**Published:** 2025-04-15

**Authors:** Cristiano Ialongo

**Affiliations:** Department of clinical pathology, University Hospital Policlinico Umberto I, Roma, Italy

**Keywords:** quality control, analytical performance specification, multivariate analysis, analytical process

## Abstract

**Introduction:**

A control chart based on Hotelling’s T^2^ multivariate statistics was used to monitor the quality of an immunoenzymatic assay for plasma levetiracetam. The chart incorporated a multi-level quality control (MLQC) system with three concentration levels of the analyte and included the analytical performance specification (APS) for therapeutic drug monitoring.

**Materials and methods:**

Data were collected from March 1 to August 14, 2024, comprising 84 consecutive triplets of values for the three MLQC levels. The initial 59 triplets were used to estimate the variance-covariance matrix and vector of means (phase I). These estimates were then applied to calculate Hotelling’s T^2^ for the remaining 25 triplets (phase II). The pharmacokinetic model of Fraser was employed to derive the APS for levetiracetam, based on a twice-daily dosing scheme and a median half-life of 8 hours.

**Results:**

The three MLQC levels showed significant correlations (r > 0.6) in both control phases. The Hotelling’s T^2^ control chart detected no out-of-specifications states (OC), compared to 12 OC signals from individual Levey-Jennings charts monitoring the MLQC levels separately. The integration of the APS into the Hotelling’s T^2^ chart provided additional insights into the process quality, and in two instances, it aligned with the OC signal from at least one of the Levey-Jennings charts.

**Conclusions:**

Hotelling’s T^2^ multivariate chart is effective for internal quality control of laboratory tests. As MLQC data offer correlated information, this approach is advantageous over multiple individual univariate charts as it ensures the correct level of false positive and false negative alarms.

## Graphical abstract

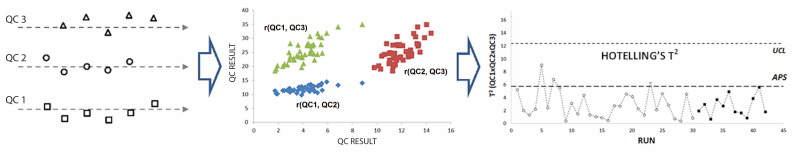

## Highlights

• Multi-level quality control controls the analytical process at different target values

• Multi-level quality control may show correlation between different quality control levels

• Hotelling’s T^2^ statistic is used to create a multivariate control chart for laboratory tests

• Multivariate control charts account for correlations between multiple quality control levels

• The Multivariate control charts are more resistant to false alarms than univariate control charts

## Introduction

Internal quality control (iQC) is an essential element of modern laboratory medicine. This statistical technique, developed in the context of industrial manufacturing by W. Shewhart in the 1930s, was introduced into clinical laboratories by Levey and Jennings in the early 1950s (*1*, *2*).

The principle of iQC is to verify the stability of the process by repeatedly sampling production over time. If the samples do not statistically differ from the assigned tendency and dispersion parameters, the process is considered to be "in-control" (IC) and remains operational; otherwise, with an error probability α (generally set at 5%), it is “out-of-control” (OC) and thus it is stopped and revised.

The fundamental tool of iQC is the control chart (CC), which is an ordered sequence or time-series plot of values from the sampled products. In order to be effective to discriminate IC from OC, the CC must have a unique target and a unique source of variation. Thereby, the current state of the process can be shown as a deviation from the target measured in respect to its natural variability.

In an analytical process, the products are represented by measurements of the biological parameter in real samples. However, under these conditions, the measurement presents a frequency distribution in relation to the differences between and within the individuals from which the samples are taken. To overcome this drawback (see Note 1 in Supplementary Material 1), the Levey-Jennings iQC removes the component of biological variability by restricting the process control to few significant values within the expected range of the measurand (*2*). To this end, it has adopted the analysis of specifically chosen or prepared samples, replicating the principle already adopted for the external quality assessment (EQA) in laboratory medicine before the iQC (*3*). This procedure, that controls the analytical process at different targets, is called multi-level quality control (MLQC).

The statistical approach to processing and analyzing Levey-Jennings iQC is univariate, as the results of each MLQC level are individually controlled (uCC). However, it should be noted that the analytical process measuring the MLQC is unique, and thus there is a potential relationship between the behavior of individual uCCs. In other words, the various levels of the biological parameter to which analytical process control is applied can be considered as different characteristics of the same product.

In industrial contexts, the correlation between different characteristics of the same product, each individually subject to quality requirements and all contributing to the quality of the final product, is well-known and has been addressed by multivariate statistical analysis introduced by H. Hotelling since the 1940s (*4*, *5*). This type of approach is logically appropriate for MLQC, and the purpose of this work is to present an application of multivariate control chart based on the Hotelling’s T^2^ statistic (mCC) to a laboratory diagnostic test.

The first part of the paper, detailed in the Materials and methods section, covers the construction of the multivariate quality control statistics T2, which is a generalization of the univariate case connected with the chi-square statistics. Readers unfamiliar with matrix algebra are encouraged to first consult Note 2 in Supplementary Material 1 for the essential concepts and terminology needed to follow the discussion. The second part, in the Results section, presents calculations based on a real laboratory dataset. An electronic spreadsheet provided in Supplementary Material 2 allows readers to simulate data and replicate the calculations, or input their own data for further exploration.

## Materials and methods

### Representation of multivariate data

The purpose of multivariate analysis is to handle MLQC using a single mCC. To understand how this is possible, think of product quality as resulting from individual quality characteristics, just as we usually perceive as a unique movement in the space the displacement of an object along each of the three directions.

It is important to note that this “unitary” treatment assumes that individual characteristics are related to each other, i.e., correlated. This raises two significant questions: 1) whether a single OC quality necessarily determines that the entire product is OC, and 2) how possible it is to correct a single characteristic without affecting the others, or rather, how plausible it is that a single characteristic can be truly OC if the others it is correlated with are not.

Consider p as the number of characteristics observed on a given product. If each one is expressed by a number of variables, then our object can be identified by a set of p-coordinates, known as a vector, always indicated by a bold symbol like as in equation (Eq.) 1 of Table 1. It is intuitive that if p = 2 or p = 3, the vector can be visualized as a point in the plane or space, respectively.

**Table 1:** Equations and formulae used for calculations

**Equation**	**Formula**	**Description**
1	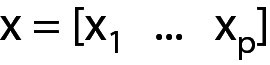	Vector of 1 observation *per* p variables
2	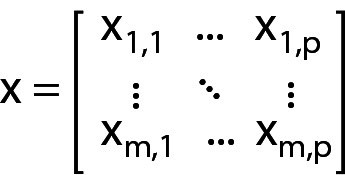	Matrix of m observations *per* p variables
3	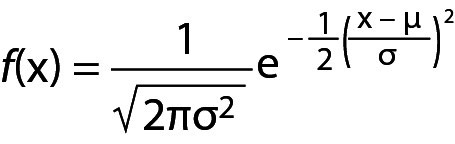	Normal univariate probability density function
4	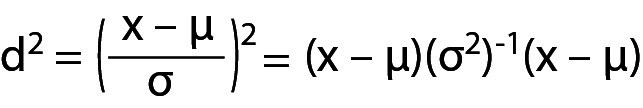	Standardized univariate quadratic distance
5	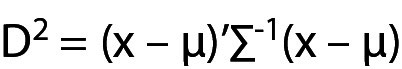	Standardized multivariate quadratic distance
6	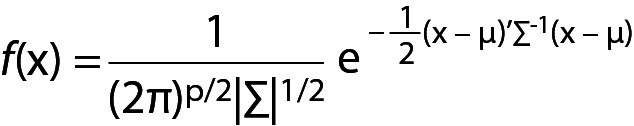	Normal multivariate probability density function
7	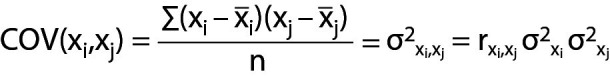	Covariance
8	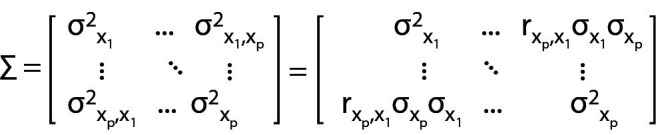	Variance-covariance matrix
9	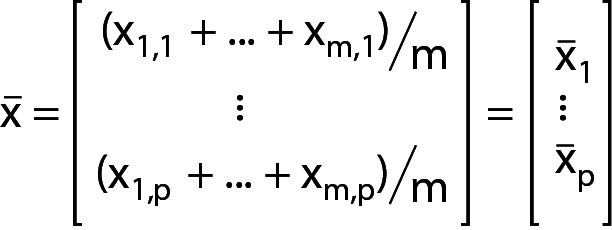	Estimate of vector of means
10	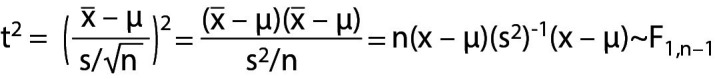	Quadratic Student’s t statistic
11	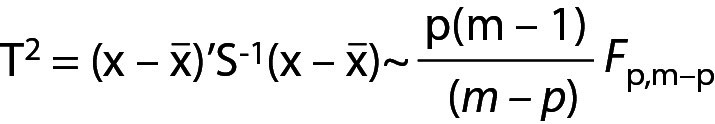	Hotelling’s T^2^ statistic
12	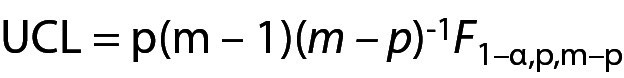	Upper control limit (UCL) of Hotelling’s T^2^-based multivariate control chart
13	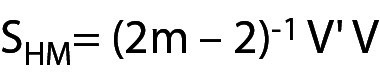	Holmes-Mergen’s estimator of variance-covariance matrix
14	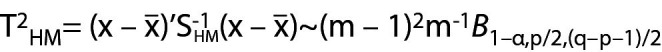	Hotelling’s T^2^ statistic with Holmes-Mergen estimator of variance-covariance matrix
Symbols: σ^2^ – population variance (parameter). p – multivariate variables. V – vector of successive overlapped differences. μ – population average (parameter). n – univariate sample size. s^2^ – sample variance (estimate). x – sample average (estimate). m – multivariate sample size. α – type I error. r_x_i_,x_j__ – correlation coefficient. B_1-α,p/2,(q-p-1)/2_ – 1-α percentile of Beta-distribution with p/2 and (q-p-2)/2 degrees of freedom, q = 2(m-1)^2^ (3m-4)^-1^. F_1-α,p,m-p_ – 1-α percentile of F-distribution with p and m-p degrees of freedom.		

If a number m of p-dimensional observations are obtained, they can be collectively represented as an array of numerical data known as a matrix (Eq. 2, Table 1). Thus, a matrix is the representation of the coordinates of a p-dimensional scatter plot, where each row is a single observation and each column represents a variable or characteristic.

### Multivariate normal distribution

The probability density function (pdf) of the normal distribution for a random variable is known to depend on the observation value, the population average *μ* and variance *σ*^2^ as in Eq. 3, Table 1. Specifically, if the exponent term is properly arranged (Eq. 4, Table 1), it yields d^2^, which measures the standardized unidimensional quadratic distance of a value from its mean.

This term is suitable for generalization to p-variables of a matrix in order to account for the correlation between them. The result is D^2^, the square of the generalized distance (Eq. 5, Table 1) (*6*). Accordingly, the pdf of the univariate normal can be extended to the multivariate case as in Eq. 6 of Table 1.

Thus, three terms are necessary to calculate D^2^ (Eq. 6, Table 1):

1. (x-μ) the difference of vectors representing the coordinates of observation and the vector of the means of p variables
2. (x-μ)' the transpose of the differences of vectors
3. Σ the variance-covariance matrix.

The Σ (Eq. 7, Table 1) has a pivotal role as it represents the structure of relationship between all the p variables, expressed as the variability of each variable both to itself as usual (variance, VAR) and to each of the other variables it is related with (covariance, COV, Eq. 8 in Table 1) (*6*).

Note that D^2^ is a scalar which means it is unidimensional, therefore the distance of the vector from the centroid of its set (or broadly to its distribution) is synthesized by a single quantity regardless of the p dimensions it captures.

### Hotelling’s T^2^ statistic and control limit

As the population parameters *μ* and *σ*^2^ can be replaced by their estimates x and s^2^ based on a sample of n univariate observations, the Student’s *t* statistic measuring the significance of a deviation from the mean can be expressed in quadratic form (t^2^) as a percentile of the *F* distribution with 1 and n-1 degrees of freedom (Eq. 9, Table 1) (*6*).

Now, replacing *μ* and Σ with their sample estimates x and S (Eq. 9, Table 1), respectively, t^2^ can be extended to the multivariate case as d^2^ to obtain for n = 1(a single replicate for each of the m multivariate observations) the Hotelling’s T^2^ statistic that follows the *F* distribution with p and m-p degrees of freedom (Eq. 11, Table 1) (*5*). Therefore, the 1-α percentile of the same distribution is the critical value for the T^2^ statistic at significance level α, *i.e.*, the upper control limit (UCL) for the mCC (Eq. 12, Table 1) (*6*).

Note that Hotelling’s mCC lacks a lower control limit (LCL): in a p-dimensional space, it does not make sense to consider a variation “above” or “below,” but only a deviation from the centroid of the set. Therefore, any deviation from the mean vector results in an increase in T^2^ regardless of the particular direction taken.

### Estimation of the variance-covariance matrix

For m individual observations (n = 1), different estimators S can be used, each having its own distribution from which to take the 1-α percentile (*7*).

Among them, the estimator by Holmes and Mergen, *S_HM_*, based on successive overlapping differences (Eq. 13, Table 1), has the advantageous characteristics of being unbiased and sensitive to both step and progressive shifts of the mean vector (*7*). This is possible as the difference between the m-th and the next observation allows retain some memory of the previous state of the process, which is commonly ignored in memoryless charts like Hotellings’ T^2^ and Shewart-type (and, by the way, the reason to implement runs rules that capture patterns and trends).

In this estimator, (2m-2)^-1^ is a constant, V is the matrix of m-1 overlapping differences, and V’ is the transpose of V (see Note 2 in Supplementary Material 1 for and explanation of transposition). When it is used *S_HM_*, *T^2^_HM_* is equal to (m – 1)^2^m^-1^ times the 1-α percentile of the beta distribution *B* with parameters p/2 and (q – p – 1)/2, where q is a constant (Eq. 14, Table 1) (*7*).

It must be noted that in industrial contexts, *S_HM_* is calculated through retrospective analysis of historical process data (HDA) collected during start-up stage or phase I of control (*5*, *8*). Actually, Levey-Jennings’ iQC represents phase II of control (*i.e.*, future control stage) as the target value and its acceptance limits are already provided by the MLQC manufacturer that conducts the phase I. As MLQC is used for uCC, this information is incomplete for the mCC as it lacks the covariance between control levels. Therefore, to compute *S_HM_*, phase II data collected in the laboratory over at least 6 months can be appropriately used as a pseudo-HDA (*9*).

## Integration of analytical performance specification

The natural limits of process variability do not necessarily coincide with the limits imposed by the diagnostic use of the test. This reference for the reliability of the test need is represented by the analytical performance specifications (APS) (*10*). In order to use the APS as adjunctive control limits within the mCC along with the UCL, we consider APS = x + δ as a tolerable deviation from the centroid of the process, where δ = dx. In vector terms:



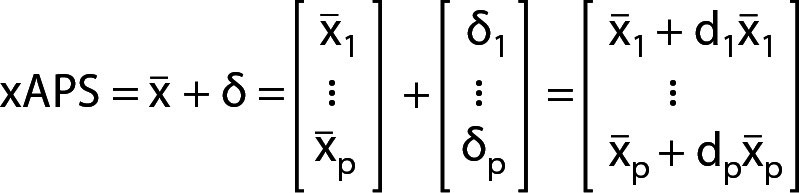



(Eq. 15).

Thus:



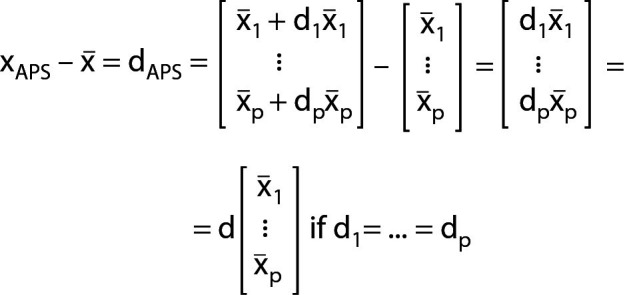



(Eq. 16),

where d is a scalar that represent the APS if the same value is used for all the p variables (otherwise, it is necessary to use the vector where each row represents the value of APS specified for the given level of the MLQC). From Eq. 16 it follows that:



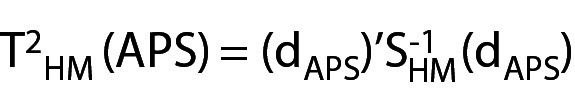



(Eq. 17).

### Data analysis

The data for generating the Hotelling’s T^2^ mCC were obtained from the analysis of the MLQC for the immunoenzymatic assay for the determination of the antiepileptic drug levetiracetam in plasma (Ark Diagnostics, Fremont, USA), performed on ILab Taurus instrumentation (Werfen, Milan, Italy) at the clinical pharmacology laboratory of the University Hospital Policlinico Umberto I of Rome.

The MLQC (provided by the same manufacturer of assay reagents) consists of p=3 control levels with target nominal concentrations of 7.5 μg/mL for QC1, 30 μg/mL for QC2, and 75 μg/mL for QC3. As batch analysis is carried out for this drug, with at least two batches *per* week of no more than 10 samples each, the MLQS is analyzed at the beginning of each run. The collected data refer to the period from March 1 to August 14, 2024, and consist of m = 84 triplets of values.

For the retrospective phase I analysis, the pseudo-HDA dataset was created with the MLQC results from the period March 1 to June 28, 2024, consisting of m = 59 triplets, and used to estimate μ (see Note 3 in Supplementary Material 1) and *S_HM_*. The data from June 1 to August 14, 2024, consisting of m = 25 triplets, were used for phase II analysis. For simplicity, in this work, it was assumed that the MLQC was centered on the phase I means and that *S_HM_* remained unchanged in phase II (consequently, and also for simplicity of presentation, the mCC for *S_HM_* was omitted).

For each phase of the analysis, the bivariate Pearson linear correlation (r) was evaluated for statistical significance using the t-test with n-2 degrees of freedom (H_0_: r = 0 *vs*. H_1_: r ≠ 0), setting P-value < α/p = 0.05 / 3 = 0.017 for multiple comparisons. An analysis of the bivariate correlation as consequence of autocorrelation in the MLQC dataset is provided in the Note 4 of Supplementary Material 1. The multivariate normality was assessed with Mardia’s test for skewness and kurtosis.

The value of APS was calculated using Fraser’s pharmacokinetic model (APS_pk_):



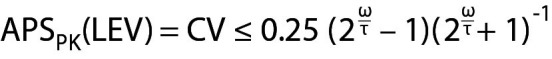



(Eq. 18),

where CV is the coefficient of variation, ω is the dosing interval, and τ is the average half-life of the drug (*11*). The minimum acceptable level was set to 1.5 times the APS (*12*).

**Figure 1:**
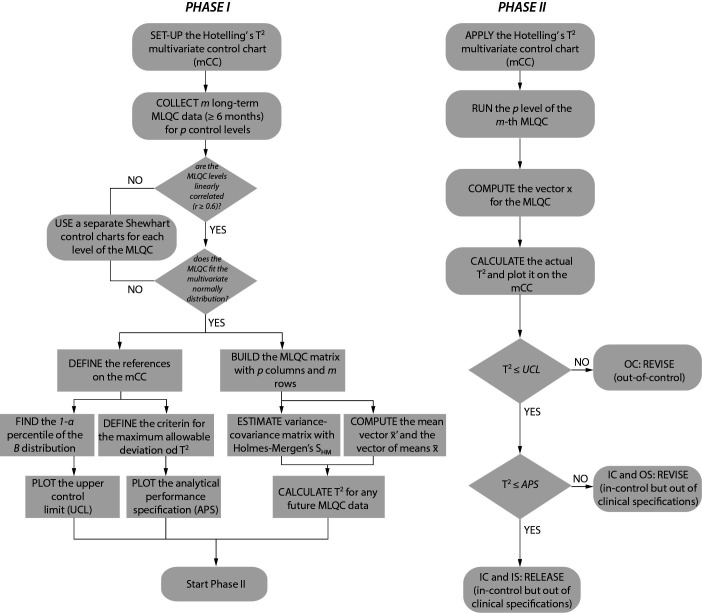
Flowchart of set-up (phase I) and application (phase II) of Hotelling’s T^2^ multivariate control chart (mCC) for the multi-level quality control (MLQC). The diagrams summarize what is described in the Materials and methods. In phase I, historical data are analyzed retrospectively to derive the variance-covariance matrix and mean vectors needed to calculate T^2^, with the aim of verifying their bivariate correlation and multivariate normality. Simultaneously, the percentile of the T^2^ distribution is calculated to establish the upper control limit (UCL), as well as the maximum acceptable deviation according to the analytical performance specification (APS). In phase II, the covariance matrix and mean vectors are used to calculate the T^2^ of the current MLQC, which is then compared with the UCL and APS values to determine the process status and to review or release the results.

All calculations were performed using Microsoft Excel 2010 (Microsoft Corporation, Redmond, USA), except for Mardia’s test, which was executed using the online tool at WebPower (*13*). An example spreadsheet with calculations is provided within Supplementary material 2 (see Note 5 for details). The flowcharts of phase I and II data analysis are outlined in Figure 1.

## Results

### Bivariate correlation and multivariate normality

Phase I and II data are summarized in Table 2. As noted, the three levels of the MLQC are correlated with each other in both phases with r > 0.6. This correlation was not an artefact of isolation of data components from an autocorrelated series, as shown in Note 4 in Supplementary Material 1.

**Table 2:** Descriptive analysis, Pearson’s bivariate linear correlation, and multivariate normality (Mardia’s test) of the multi-level quality control data for the for the levetiracetam assay in phase I (pseudo-HDA) and phase II of real-time quality control

	**Phase I**			**Phase II**			
Sample size (triplets, m)		59			25		
	**QC1 (ng/mL)**	**QC2 (ng/mL)**	**QC3 (ng/mL)**	**QC1 (ng/mL)**	**QC2 (ng/mL)**		**CQ3 (ng/mL)**
Average	8.796	31.950	80.249	8.535	31.389		81.742
Standard deviation	0.729	2.347	4.198	0.651	2.646		4.203
CV (%)	8.3	7.3	6.1	7.6	8.4		5.1
	r (QC1,QC2)	r (QC1,QC3)	r (QC2,QC3)	r (QC1,QC2)	r (QC1,QC3)		r (QC2,QC3)
Linear correlation (Pearson)	0.749*	0.719*	0.617*	0.789*	0.630*		0.739*
	b	z	P-value	b	z		P-value
Mardia’s test of multivariate kurtosis	0.827	8.129	0.616	1.316	5.265		0.873
Mardia’s test of multivariate skewness	15.066	0.046	0.963	12.964	- 0.910		0.363
*P-value < 0.017 (H_0_: r = 0 *vs.* H_1_: r ≠ 0)							

Similarly, the datasets used for phase I and phase II analyses do not significantly deviate from multivariate normality.

### Mean vector and covariance matrix

The vector of means x and its transpose x’ estimated from the pseudo-HDA data are the following:



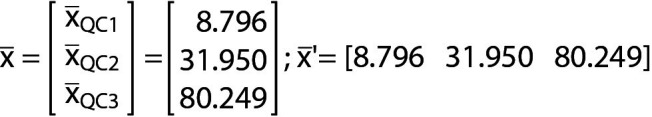



(Eq. 19).

For the estimation of *S_HM_* and its inverse *S_HM_^-1^*, the results are:



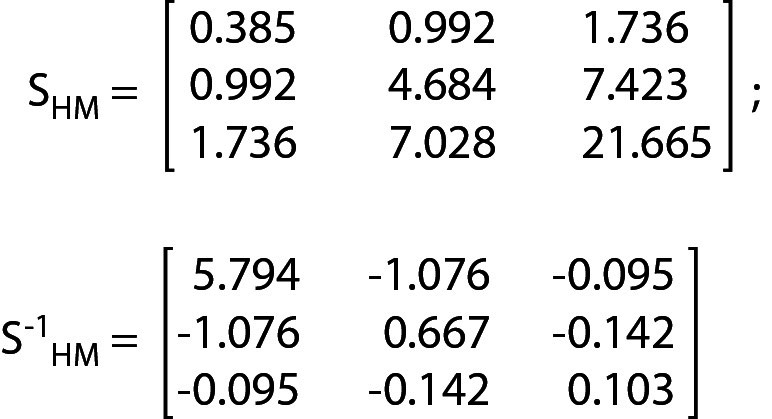



(Eq. 20).

### Upper control limit

The percentile 1 − α of *B*-distribution for α = 0.05, p = 3, and q = 39 corresponds to:



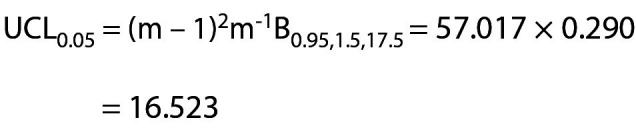



(Eq. 21).

This represents the limit value for the chart relative to the running state (*i.e.*, within ±2 sd of the univariate chart). Therefore, if T_2HM_ > 11.301, the analytical process is OC.

### Analytical performance specifications

Based on Eq. 18, with a dosing frequency of twice daily (ω = 12) and a median half-life of 8 hours (τ = 8), the APS_pk_(LEV) is calculated to be 0.12. Substituting this value into Eq. 16 yields a d_APS_ for minimum acceptability equal to:



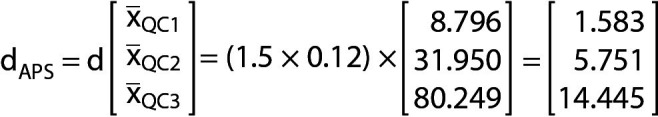



(Eq. 22).

Thus, by performing the calculations as in Eq. 17 it yields:



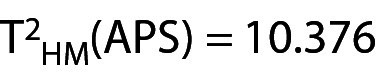



(Eq. 23).

If T^2^_HM_ > 10.376, the analytical process is outside the clinically acceptable specifications *i.e.* “out-of-specification” (OS); however, if 10.376 < T^2^_HM_ < 16.523, the analytical process is IC but OS.

### Hotelling’s T^2^ mCC and comparison with Levey-Jennings uCC

**Figure 2:**
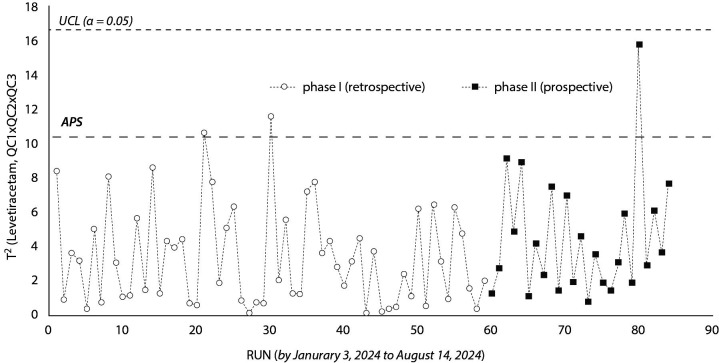
Hotelling’s T^2^ multivariate control chart (mCC) for the multi-level quality control (MLQC) of the levetiracetam assay. The mCC displays the run number on the x-axis and the Hotelling’s T^2^ statistic on the y-axis, which measures the deviation of the vector resulting from the three levels of the MLQC from the vector of means (*i.e.*, the centroid of the multivariate normal distribution of the data). In phase I, the MLQC data are analyzed retrospectively to find the vector of means, the variance-covariance matrix, the upper control limit (UCL), and the analytical performance specification (APS) of the mCC (respectively Eq. 19, Eq. 20, Eq. 21, and Eq. 23 in the text); in phase II the information is used to control the state of the analytical process.

Table 3 summarizes the OC conditions reported by Hotelling’s T^2^ mCC and the three Levey-Jennings uCCs for the MLQC. It is noted that the mCC signalled no OC, even when it occurred in more than one uCC simultaneously. In two cases (one *per* each phase of control), the OS condition corresponds to the OC state for uCCs of QC2 and QC3. Figure 2 shows the mCC for phase I and phase II data.

**Table 3:** Out-of-control and out-of-specification conditions with attributable cause indicated by Hotelling’s T^2^ multivariate control chart and Levey-Jennings univariate control charts for the levetiracetam assay

		**Hotelling CC**			**Levey-Jennings CC**			
	**run**	**T^2^**	**> APS**	**> UCL**	**< LCL or > UCL**			
					**QC1**	**QC2**	**QC3**	**Attributable cause**
	16	4.468					<	gross/random error
	21	10.636	>		>	>		carryover/dirty cuvettes
	22	7.836				>	>	reagent decay (lot change)
Phase I	24	5.157					<	gross error/QC aliquot degradation
	30	11.576	>					carryover/dirty cuvettes (?)
	36	7,804			<			QC aliquot degradation
	52	6.504				>	>	carryover/cuvettes cleaning
	55	6.321			>			QC aliquot degradation
	62	9.146			<	<		QC aliquot degradation (lot change)
Phase II	72	4.907				>		gross error/QC aliquot degradation
	78	5.953					>	QC aliquot degradation
	80	15.772	>			<		gross error (needle obstruction)
CC - control chart. APS - analytical performance specification. UCL - upper control limit. LCL - lower control limit. QC - quality control level.								

## Discussion

The use of the multivariate model is aimed at aligning the statistical control tool with the structure of the analytical process, which shows correlated control levels. Correlation, in fact, indicates an associative relationship in the data, with a functional explanation in the shared calibration, reagents, volumes, and measurement instruments used for all analyses performed for the same test.

**Figure 3:**
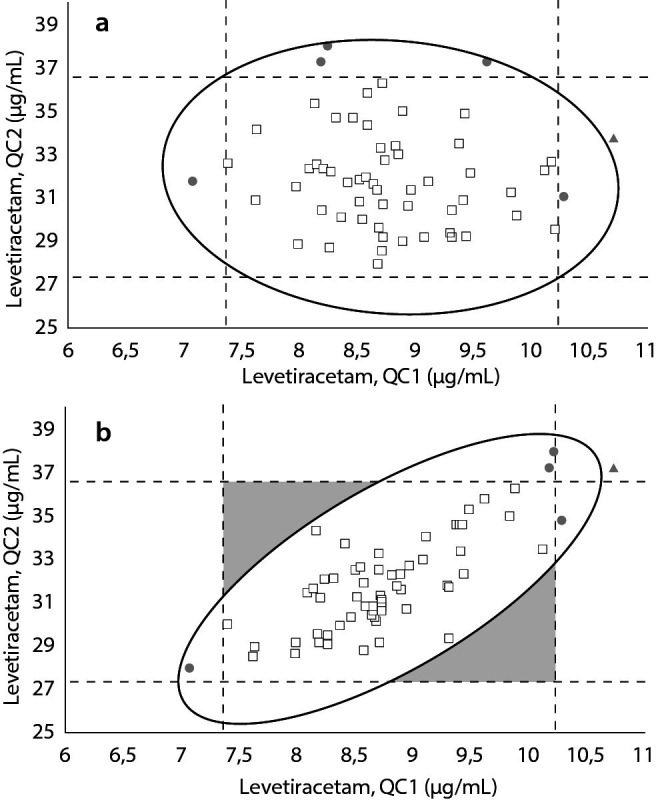
Scatterplot of level 1 of quality control (QC1) *versus* level 2 (QC2) of the multi-level quality control (MLQC) of the levetiracetam assay. Panel “a” shows the MLQC data for phase I of QC1 and QC2 along with their corresponding univariate control levels (gray dashed lines). The QC2 data we shuffled to remove correlation with QC1. The overlap of these lines creates the rectangular pseudo-bivariate control area (PBCA), outside of which lie the out-of-control (OC) values of the process. In the same panel, the solid black circle identifies the truly bivariate control area (BCA), outside of which lie the out-of-control (OC) values of the process. The overlap between BCA and PBCA is maximum in this case. Panel ‘b’ shows the same data with correlation (r = 0.78). As a result, the BCA takes the elliptical shape like data (solid black line) minimizing its overlap within the PBCA, as shown by the dashed-grey areas. Filled triangles represent OC for both BCA and PBCA, while filled circles represent OC for PBCA only.

From a quantitative perspective, correlation and autocorrelation of control data (see Note 4) result in a decline in the performance of uCC in identifying true (power) and false (specificity) OC conditions (*5*, *14*). This manifests as a delay in the detection of analytical errors by a single uCC. In the case of multiple uCCs, commonly used in MLQC control, there is also an inflation of α, arising from the combined use of univariate control limits, as explained in Figure 3. Therefore, in the presence of correlation or autocorrelation, analytically estimated performance is falsely better than actual performance.

The mCC is an extension of the uCC, where the correlational structure of the process is used as a tool to measure the degree of deviation in the p characteristics that compose it (*4*). Consequently, similar to the uCC, the power of the chart decreases as the analytical error drops below the multivariate equivalent of 1.5 sd (*15*, *16*). Unlike the uCC, the use of the correlation structure makes the performance of the mCC dependent on several additional factors: the choice of *S* and its accuracy, the size of p and n, the concordance of signs among errors at the levels, and the concordance between these signs and the correlation of the levels where they occur (*7*, *15*, *17*).

**Figure 4:**
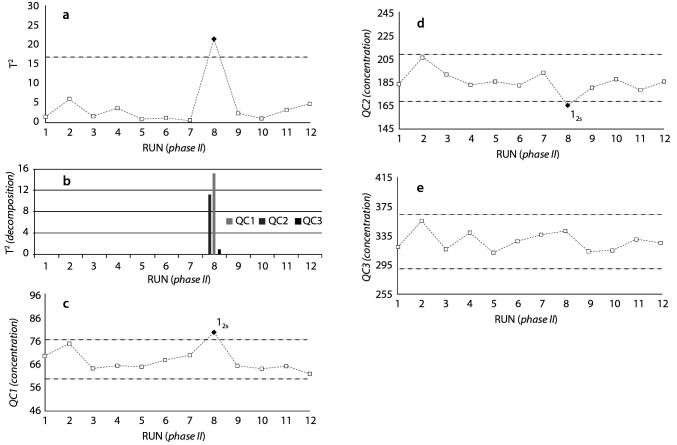
Hotelling’s T^2^ multivariate chart (mCC) and Decomposition of T^2^ *vs.* R_4s_ “across runs” out-of-control (OC) in multiple Levey-Jennings univariate control charts (uCC) in the presence of a 2 sd shift with opposite signs in 2 out of 3 levels (QC1, QC2) of multi-level quality control (MLQC). The mCC (panel “a”) indicates an OC at run 9, which the decomposition of T^2^ attributes to QC1 and QC2 (panel “b”); the univariate control charts uCC for QC1 (panel “c”) and QC2 (panel “d”) show an OC for isolated 1_2s_ signals, which, having opposite signs, jointly generate an OC R_4s_ “across runs”; the uCC for QC3 (panel “e”) does not indicate any OC. The data were simulated to obtain correlation r (QC1,QC2) = 0.78, r (QC2,QC3) = 0.70, and r (QC1,QC3) = 0.62, with a precision of CV% (QC1) = 15.0, CV% (QC2) = 10.0, CV% (QC3) = 7.0. The control limits are represented by the horizontal dashed line and are set for α = 0.05.

**Figure 5:**
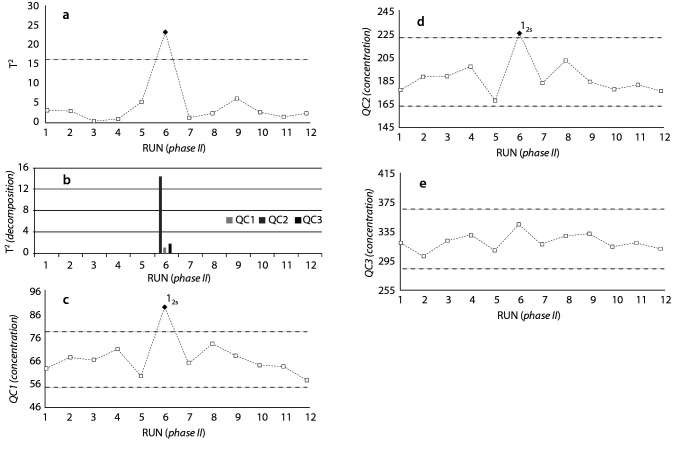
Hotelling’s T^2^ multivariate chart (mCC) and decomposition of T^2^ vs. 2_2s_ “across runs” out-of-control (OC) in multiple Levey-Jennings univariate control charts (uCC) in the presence of a 2sd shift with the same sign in 2 out of 3 levels (QC1, QC2) of multi-level quality control (MLQC). The mCC (panel “a”) indicates an OC at run 6, which the decomposition of T^2^ attributes to QC1 but not to QC2 (panel “b”); the uCC for QC1 (panel “c”) and QC2 (panel “d”) show an OC for isolated 1_2s_ signals, which, having the same sign, jointly generate an OC 2_2s_ “across runs”; the uCC for QC3 (panel “e”) does not indicate any OC. The data were simulated to obtain correlation r (QC1,QC2) = 0.78, r (QC2,QC3) = 0.70, and r (QC1,QC3) = 0.62, with a precision of CV% (QC1) = 15.0, CV% (QC2) = 10.0, CV% (QC3) = 7.0. The control limits are represented by the horizontal dashed line and are set for α = 0.05.

The impact of these factors must necessarily be considered in the application scenario, because the sign and magnitude of the error in the p levels depend on the specific structure of the analytical process (*18*, *19*). In modern clinical chemistry, where most of the sample processing in automated, systematic errors tend to prevail and this is congenial to the sensitivity and robustness of the *S_HM_* (*7*). Considering that with p ≤ 3 and n ≤ 2, which reflects the most probable MLQC scenario, the accurate choice of the estimator becomes crucial as power of the mCC decreases with concordant components of error in the p levels (*15*). The components of errors in the p levels of the MLQC give rise to different “across runs” control schemes in the univariate model: R_4s_ for discordant errors (Figure 4), 2_2s_ for concordant ones (Figure 5) (*20*).

**Figure 6:**
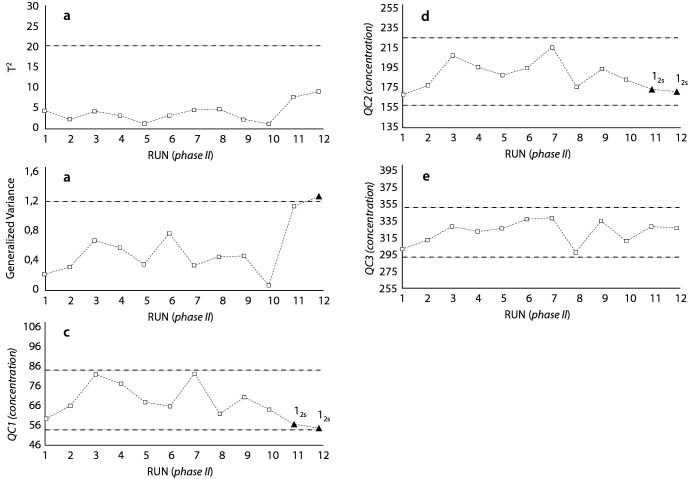
Hotelling’s T^2^ multivariate chart (mCC) and generalized variance chart (GV) vs. 41s “across runs” out-of-control (OC) in multiple Levey-Jennings univariate control chart (uCC) in the presence of a 1sd shift with the same sign in 2 out of 3 levels (QC1, QC2) of multi-level quality control (MLQC). The mCC, (panel “a”) indicates no OC, however the GV (panel “b”) shows a value at the control limit at run 11 and an OC at run 12; the uCC for QC1 (panel “c”) and QC2 (panel “d”) show consecutive 1_1s_ signals at runs 11 and 12, which, having the same sign, jointly generate an OC 4_1s_ “across runs”; the uCC for QC3 (panel “e”) does not participate in the formation of the “across runs” pattern. The data were simulated to obtain correlation r (QC1,QC2) = 0.78, r (QC2,QC3) = 0.70, and r (QC1,QC3) = 0.62, with a precision of CV% (QC1) = 15.0, CV% (QC2) = 10.0, CV% (QC3) = 7.0. The control limits are represented by the horizontal dashed line and are set for α = 0.05.

The synthesis of p levels into a single statistic is a defining feature of this analytical tool, along with a single control level. For correlated data, this avoids redundancy in MLQC patterns across uCCs, facilitating the interpretation of the process state, especially when applying runs rules to the mCC (*21*). If the process is OC, the significantly deviant components among the p levels can be identified using the decomposition of T^2^ as illustrated in Figures 4 and 5 (*22*, *23*). Since this technique is applied only when the value is statistically significant for the underlying multivariate model, it is in principle a *post-hoc* test that controls the inflation of α. Therefore, it cannot be compared with the use of multiple uCCs or “across runs” rules that assume univariate and independent data (see what described in Figure 2 and the results in Figure 5 and Figure 6). Furthermore, as a standardized variable, T^2^ can be used directly for performance comparisons between processes (similar to the two-sample t-test) or against a reference value, as proposed in this study with the APS, whose results are shown in Table 3.

From this demonstrative and didactic approach arise the two main simplifications adopted in this study. The first is the use of a pseudo-HDA instead of a statistically-planned phase I, calculating m based on the desired power for OCs. In fact, the sample size is larger for both p and n small, so in clinical chemistry it is expected to be m > 100 (*15*, *17*). Therefore, the mere temporal criterion of covering the variability of the analytical process does not guarantee *per se* the accuracy of the estimator and, consequently, desired control performance. The second simplification assumes the stability of the process correlation structure to avoid introducing an additional multivariate tool, namely the generalized variance chart. While this helps the reader focus on the basics of multivariate analysis, it prevents a rigorous verification of the assumption of consistency between phase I and phase II of the mCC structure. Moreover, especially when m = 1, the change in *S* delivers information on the state of control of the process as some kind of errors tend to alter the structure of correlation.

Collectively, these limitations make the application results of this study, such as those in Table 3, valid only as proof of the feasibility of the methods discussed. The reader is invited to take them into careful consideration if aiming at replicating this experience, and mostly, when considering the results in Table 3. Indeed, in the absence of a rigorous statistical performance analysis, the opposing behavior of the two control systems in terms of reported OC cannot serve as evidence of greater specificity or lower power of the mCC. Actually, it only proves the existence of a difference and the need to investigate it further analytically.

A final consideration concerns the arbitrariness of the test chosen as the model for applying the mCC. This work was motivated by the impression of some degree of redundancy in MLQC patterns observed during routine inspection of uCCs. The incidental discovery of correlation, rather than its systematic investigation in analytical quality data, demonstrates that this phenomenon, its consequences, and the tools to control it, are not part of the laboratory professional’s knowledge base and quality routine. It suffices to note that references on multivariate quality in laboratory medicine in the literature are few and confined to a timespan of a decade (*19*, *24*-*26*). Whether this reflects the marginality of the subject is difficult to say. However, the methodological complexity imposed by multivariate methods cannot serve as a valid excuse if, as J.H. Livesey aptly stated in 2005, “now, since digital computers are almost universally available, it is more efficient to base QC procedures on the most powerful and selective statistical algorithms available” (*26*).

In conclusion, this work demonstrates the feasibility of implementing Hotelling’s T^2^ mCC for iQC in a laboratory test where the correlation between MLQC levels has been proven. The chart, which allows monitoring a single statistic against multiple control levels, has the potential to streamline the management of laboratory analytical processes. To put this approach into practice, it is essential to recognize it as an evolution of the quality paradigm beyond the single dimension drawn by Levey and Jennings. This is possible, if not necessary, in an era that actively promotes the statistical expertise of clinical laboratory specialists and gives them means to leave the comfort zone of univariate concepts.

## Data Availability

The data generated and analyzed in the presented study are available from the corresponding author on request.
